# Altered Intra- and Interregional Synchronization in Resting-State Cerebral Networks Associated with Chronic Tinnitus

**DOI:** 10.1155/2015/475382

**Published:** 2015-02-05

**Authors:** Yu-Chen Chen, Jian Zhang, Xiao-Wei Li, Wenqing Xia, Xu Feng, Cheng Qian, Xiang-Yu Yang, Chun-Qiang Lu, Jian Wang, Richard Salvi, Gao-Jun Teng

**Affiliations:** ^1^Jiangsu Key Laboratory of Molecular and Functional Imaging, Department of Radiology, Zhongda Hospital, Medical School, Southeast University, Nanjing 210009, China; ^2^Center for Hearing and Deafness, University at Buffalo, Buffalo, NY 14214, USA; ^3^Department of Physiology, Southeast University, Nanjing 210009, China; ^4^Medical School, Southeast University, Nanjing 210009, China; ^5^Center for Functional Neuroimaging, University of Pennsylvania, Philadelphia, PA 19104, USA; ^6^Department of Otolaryngology, Zhongda Hospital, Medical School, Southeast University, Nanjing 210009, China; ^7^School of Human Communication Disorders, Dalhousie University, Halifax, NS, Canada B3J 1Y6

## Abstract

*Objective*. Subjective tinnitus is hypothesized to arise from aberrant neural activity; however, its neural bases are poorly understood. To identify aberrant neural networks involved in chronic tinnitus, we compared the resting-state functional magnetic resonance imaging (fMRI) patterns of tinnitus patients and healthy controls. *Materials and Methods*. Resting-state fMRI measurements were obtained from a group of chronic tinnitus patients (*n* = 29) with normal hearing and well-matched healthy controls (*n* = 30). Regional homogeneity (ReHo) analysis and functional connectivity analysis were used to identify abnormal brain activity; these abnormalities were compared to tinnitus distress. *Results*. Relative to healthy controls, tinnitus patients had significant greater ReHo values in several brain regions including the bilateral anterior insula (AI), left inferior frontal gyrus, and right supramarginal gyrus. Furthermore, the left AI showed enhanced functional connectivity with the left middle frontal gyrus (MFG), while the right AI had enhanced functional connectivity with the right MFG; these measures were positively correlated with Tinnitus Handicap Questionnaires (*r* = 0.459, *P* = 0.012 and *r* = 0.479, *P* = 0.009, resp.). *Conclusions*. Chronic tinnitus patients showed abnormal intra- and interregional synchronization in several resting-state cerebral networks; these abnormalities were correlated with clinical tinnitus distress. These results suggest that tinnitus distress is exacerbated by attention networks that focus on internally generated phantom sounds.

## 1. Introduction

Subjective tinnitus, a phantom sound, is often described as a ringing, hissing, or buzzing sensation [1]. In the United States, an estimated 50 million adults have experienced tinnitus occasionally and 16 million experience it chronically [2]. Patients with chronic tinnitus often suffer from sleep disturbance, depression, and anxiety, conditions that negatively impact the quality of life [3]. Since tinnitus often persists even after sectioning the auditory nerve [4] and since tinnitus masking profiles differ from external sounds [5], aberrant neural activity in the central nervous system (CNS) rather than the cochlea is believed to play a major role in its development and maintenance [6–8]. On the basis of previous electrophysiological and neuroimaging studies [9, 10], tinnitus is believed to be generated by aberrant neural activity in the central auditory pathway by a variety of mechanisms such as increased spontaneous activity, increased neural synchrony, altered tonotopy, aberrant neural connectivity between auditory and nonauditory structures, aberrant attentional, or gating mechanisms [11]. The fact that some patients are constantly aware of their tinnitus and find it extremely disturbing suggests that attentional or emotional neural networks may contribute to the severity of tinnitus distress [12].

Since subjective tinnitus is an endogenous ongoing process, resting-state functional magnetic resonance imaging (fMRI) has proved to be a useful noninvasive technique for determining how structurally segregated and functionally specialized cerebral centers are interconnected as reflected by low-frequency (0.01–0.1 Hz) fluctuations in blood-oxygenation-level dependent (BOLD) signals [13–15]. Previous studies have employed resting-state fMRI to examine multiple whole-brain networks such as the attention network and default mode network (DMN) to study the neural mechanisms of tinnitus [16]. Kim et al. found increased functional connectivity between the resting-state attention network and an auditory network suggesting that this network might contribute to the perception or salience of tinnitus [17]. Burton et al. proposed that tinnitus associated with hearing loss was the result of usage-induced changes in the organization of sensory networks and interference with the ventral and dorsal attention networks and executive control of attention (ECA) networks [12]. Because of its persistent nature, some have proposed that tinnitus generators become integrated with the DMN [18, 19]. Schmidt et al. also identified tinnitus-specific alterations in the connectivity encompassing the DMN and dorsal attention network [20]. Therefore, the above studies suggest that the attention network as well as the DMN may play a pivotal role in the neurological pathophysiology of tinnitus. Moreover, our group found that chronic tinnitus patients with normal hearing out to the extended high frequencies exhibited aberrant amplitude of low-frequency fluctuations (ALFF) in specific DMN regions including the middle temporal gyrus (MTG) and angular gyrus; the magnitude of the ALFF alterations was correlated with tinnitus severity [21].

One limitation of previous tinnitus studies is that they mainly measured the interregional synchronization between distinct brain areas or correlations between resting-state connectivity networks while few have focused on investigating abnormal intraregional activity in specific brain regions. Regional homogeneity (ReHo) analysis, a robust algorithm that quantifies the local synchronization within neighboring voxels during resting state [22], can be used to identify aberrant local neural activity coherence across the whole brain compared with the functional connectivity analyses. Altered ReHo values may relate to disequilibrium of spontaneous neural activity within and between corresponding brain regions. Indeed, aberrant ReHo values, indicative of disrupted local functionality, have been linked to several neurological impairments such as Alzheimer's disease, Parkinson's disease, schizophrenia, and hepatic encephalopathy [23–26]. Therefore, ReHo measurement is a potentially powerful tool to detect aberrant resting-state brain activity which complements information provided by functional connectivity analysis.

In this study, we explored the intra- and interregional synchronization of multiple cerebral networks by combining ReHo and functional connectivity analyses to gain additional insights regarding aberrant neural activity in chronic tinnitus patients with normal hearing thresholds. We speculated that (1) abnormal ReHo and functional connectivity would be detected within tinnitus-related networks such as the attention network and DMN and (2) these abnormalities in the resting-state networks would be correlated with specific tinnitus characteristics such as tinnitus distress.

## 2. Materials and Methods

### 2.1. Subjects and Study Design

Thirty chronic tinnitus patients and 30 healthy subjects without tinnitus were recruited through newspaper advertisements and community health screenings from September 2011 to September 2013. All participants were right-handed and completed at least 8 years of education. One of the tinnitus patients was subsequently excluded from the study because of the exceeded limits for head motion during MR scanning. The tinnitus patients and healthy subjects were group-matched with respect to age, sex, and education. Twelve patients reported predominantly left-sided tinnitus, 6 reported predominantly right-sided tinnitus, and 11 described their tinnitus as bilateral or originating within the head. The severity of tinnitus and related distress were assessed by the Iowa version of the Tinnitus Handicap Questionnaires (THQ) [27]. Hearing threshold was determined by pure tone audiometry (PTA) examination. All the participants had no hearing loss in any of 10 measured audiometric frequencies ranging from 250 Hz to 16 kHz (hearing thresholds < 25 dB). There were no significant differences in auditory thresholds between tinnitus and control groups. None of the participants had symptoms of depression or anxiety based on the Self-Rating Depression Scale (SDS) and Self-Rating Anxiety Scale (SAS) (overall scores < 50) [28, 29]. Participants were excluded if they reported suffering from hyperacusis, Meniere's diseases, or pulsatile tinnitus or if they had a past history of heavy smoking, alcoholism, stroke, head injury, Parkinson's disease, Alzheimer's disease, major depression, epilepsy, or other neurological or psychiatric illness, major medical illness (e.g., anemia, thyroid dysfunction, and cancer), MRI contraindications, and severe visual impairment.  Table 1 summarizes the characteristics of the chronic tinnitus patients and healthy subjects. This study was approved from the Research Ethics Committee of the Affiliated Zhongda Hospital of Southeast University. Written informed consent was obtained from all subjects.

### 2.2. MRI Scanning

MRI data were acquired using a 3.0-Tesla Trio scanner (Siemens, Erlangen, Germany). Foam padding and earplugs were used to reduce head motion and scanner noise. The earplugs (Hearos Ultimate Softness Series, USA) were used to attenuate scanner noise by approximately 32 dB. Subjects were instructed to keep their eyes closed without falling asleep, not to think of anything in particular, and to avoid any head motion during the scan. Structural images were obtained using a T1-weighted 3D spoiled gradient-echo sequence (repetition  time = 1900 ms; echo  time = 2.48 ms; thickness = 1 mm; gap⁡ = 0 mm; acquisition  matrix = 256 × 256; slices = 176; flip  angle = 90°; field  of  view = 250 mm × 250 mm). Functional images were acquired using a gradient-echo planar sequence (repetition  time = 2000 ms; echo  time = 25 ms; thickness = 4 mm; gap⁡ = 0 mm; acquisition  matrix = 64 × 64; slices = 36; flip  angle = 90°; field  of  view = 240 mm × 240 mm). The resting state recording took 8 minutes and 6 seconds.

Whole-brain volumes were calculated by using the VBM8 toolbox (http://dbm.neuro.uni-jena.de/vbm/). Briefly, the unified segmentation model was used to segment cerebral tissues into gray matter (GM), white matter (WM), and cerebrospinal fluid (CSF). GM and WM volumes were calculated by estimating these segments. Total brain parenchyma volume was computed as the sum of GM and WM volumes.

### 2.3. Data Preprocessing

Functional data were preprocessed with the toolbox Data Processing Assistant for Resting-State fMRI programs [30] through Statistical Parametric Mapping (SPM8; http://www.fil.ion.ucl.ac.uk/spm/) and resting-state fMRI data analysis toolkit (REST1.8; http://www.restfmri.net/). The first 10 volumes were discarded and the remaining 230 consecutive volumes were used for data analysis. Afterwards, the procedures were carried out as follows: slice-timing adjustment, realignment for head-motion correction, spatially normalized into the stereotactic space of the Montreal Neurological Institute (MNI) (resampling voxel size = 3 × 3 × 3 mm^3^) and smoothed using a Gaussian kernel of 4 mm full width at half-maximum (FWHM), detrending, and filtering (0.01–0.08 Hz). The participants who had a head motion more than 2.0 mm displacement or a 2.0° rotation in the *x*, *y*, or *z* directions were excluded from this study.

### 2.4. ReHo Analyses

ReHo analyses were calculated using REST software. Briefly, Kendall's coefficient of concordance (KCC) was computed to measure the local synchronization of a given voxel with 26 nearest neighboring voxels in a voxelwise way [22], and the ReHo value was assigned to the central voxel. The procedures used to acquire individual ReHo maps were implemented using REST software. Individual ReHo maps were divided by the global mean value within the whole brain mask for normalization. Then, the normalized ReHo maps were spatially smoothed with a Gaussian kernel of 4 mm FWHM. A two-sample *t*-test was performed on the group ReHo maps in a voxel-by-voxel way to explore the ReHo differences between two groups. A threshold was corrected *P* < 0.01, with a Monte Carlo simulation for multiple comparisons (http://afni.nimh.nih.gov/pub/dist/doc/manual/AlphaSim.pdf/). Age, sex, education, and GM volume were included as nuisance covariates, to control for the possible influences of these four factors on the results.

### 2.5. Functional Connectivity Analyses

The functional connectivity analyses were also performed using REST software. Four areas based on the increased ReHo findings were defined as regions of interest (ROI), including the bilateral anterior insular cortex (AI), the left inferior frontal gyrus (IFG), and the right supramarginal gyrus (SMG). The mean time series of each ROI was acquired for reference time course. Then, Pearson's correlation coefficients were computed between the mean signal change of each ROI and the time series of each voxel. Finally, the correlation coefficients were converted into *z* values using Fisher *z*-transform to improve the normality [31]. Six parameters of head motion and average time courses of global, WM, and CSF signals were removed by linear regression analysis.

The individual *z* values were entered into random effect one-sample *t*-tests to identify the brain regions exhibiting significant connectivity to each ROI at a threshold of *P* < 0.01 (AlphaSim correction). Two-sample *t*-tests were performed to explore the differences of the functional connectivity of each ROI between tinnitus patients and controls. A mask was created for two-sample *t*-tests by combining the regions exhibiting significant connectivity to each ROI. Age, sex, education, and GM volume were included as nuisance covariates. The threshold was also set at *P* < 0.01 (AlphaSim correction). The brain connectivity graphs were visualized using the BrainNet viewer [32] (http://www.nitrc.org/projects/bnv/).

### 2.6. Statistical Analyses

Between-group *t*-test and *χ* ^2^-test were used to compare demographic and clinical data (*P* < 0.05 was considered to be significant). To investigate the relationship between fMRI data and clinical characteristic of tinnitus patients, the regions showing significant increased ReHo or functional connectivity between tinnitus patients and healthy controls were extracted. Mean ReHo or *z* values within these clusters were correlated against each clinical characteristic of tinnitus patients using Pearson's correlation analysis by SPSS software (version 18.0; SPSS, Chicago, IL, USA). Statistical threshold was set at *P* < 0.05. Partial correlations were calculated after correction for age, sex, education, and GM volume. Bonferroni correction for multiple comparisons was applied in the correlation analyses.

## 3. Results

### 3.1. Structural Analyses

Table 2 compares the brain volumes of the chronic tinnitus patients and healthy controls. The GM, WM, and brain parenchyma volumes in subjects with tinnitus were not significantly different from healthy controls.

### 3.2. ReHo Analyses

**Figure 1:**
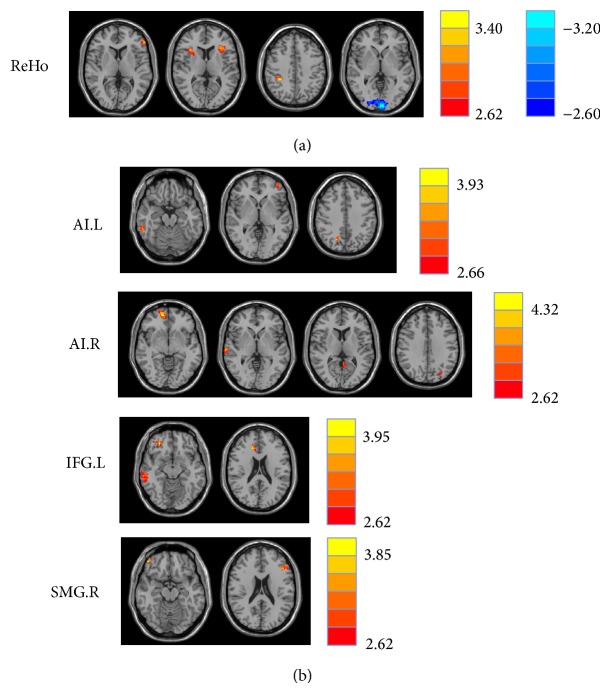
(a) Regions of significant ReHo differences in tinnitus patients compared with healthy controls. Heat map (upper, right) shows areas of increased ReHo (*t* values 2.62 to 3.40; red to yellow, resp.) and decreased ReHo (*t* values −2.60 to −3.20; dark blue to light blue, resp.). Table 3 identified regions where significant increases and decreases occurred. (b) Significant increased functional connectivity of four seed regions between tinnitus patients and healthy subjects. Table 4 identified regions where significant increases occurred. The threshold was set at *P* < 0.01 (AlphaSim correction). L: left; R: right; ReHo: regional homogeneity; AI: anterior insula; IFG: inferior frontal gyrus; SMG: supramarginal gyrus.

Compared to the healthy controls, chronic tinnitus patients had significantly increased ReHo values in the bilateral AI, left IFG, and right SMG. In contrast, decreased ReHo values in tinnitus patients were observed in the left cuneus (Figure 1(a) and Table 3).

### 3.3. Functional Connectivity Analyses

**Figure 2:**
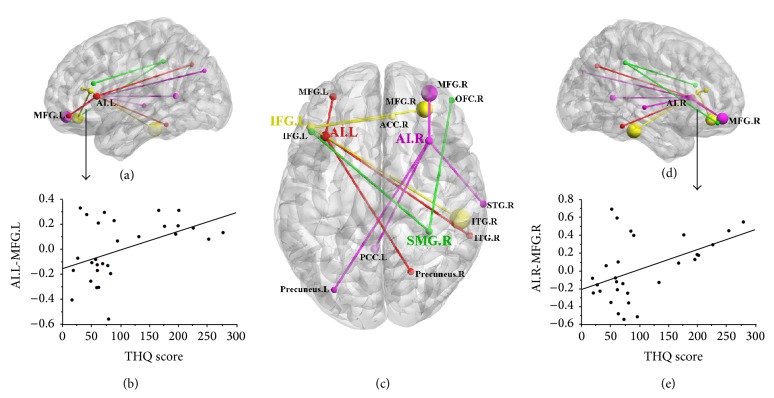
Relationships between abnormal functional connectivity and tinnitus distress. ((a)-(b)) The functional connectivity of left AI to left MFG was correlated with the THQ score (*r* = 0.459, *P* = 0.012). (c) The aberrant brain connectivity networks from four seed regions. The red represents the left AI; the magenta represents the right AI; the yellow represents the left IFG; the green represents the right SMG. ((d)-(e)) The functional connectivity of right AI to right MFG was correlated with the THQ score (*r* = 0.479, *P* = 0.009). L: left; R: right; THQ: Tinnitus Handicap Questionnaires; AI: anterior insula; IFG: inferior frontal gyrus; SMG: supramarginal gyrus; MFG: middle frontal gyrus; STG: superior temporal gyrus; ITG: inferior temporal gyrus; ACC: anterior cingulate cortex; PCC: posterior cingulate cortex; OFC: orbitofrontal cortex.

Based on increased ReHo values in tinnitus patients, we defined four ROIs for our connectivity analysis: left AI, right AI, left IFG, and right SMG. Tinnitus patients compared to controls showed significantly greater connectivity between the seed region in left AI and the left MFG, right inferior temporal gyrus (ITG), and right precuneus (Figures 1(b) and 2(c) and Table 4). Relative to controls, tinnitus patients showed significantly greater connectivity between the seed region in right AI and the right MFG, right superior temporal gyrus (STG), left precuneus, and left posterior cingulate cortex (PCC). Tinnitus patients relative to controls also demonstrated significantly increased connectivity between the seed region in left IFG and the right MFG, right ITG, and right anterior cingulate cortex (ACC). Finally, tinnitus patients compared to controls exhibited significantly greater connectivity between the seed region in the right SMG and the left IFG and right orbitofrontal cortex (OFC).

### 3.4. Correlation Analyses

In the tinnitus patients, there were no significant correlations between abnormal ReHo values and any of the tinnitus characteristics. However, the change in functional connectivity between the left AI and the left MFG was positively correlated with the THQ score (*r* = 0.459, *P* = 0.012) (Figure 2). In addition, the increased functional connectivity between the right AI and the right MFG was also positively correlated with the THQ score (*r* = 0.479, *P* = 0.009). None of the other regions of increased functional connectivity were significantly correlated with THQ scores. Moreover, none of the regions of increased functional connectivity were correlated with SDS or SAS scores or tinnitus duration.

## 4. Discussion

This present study investigated for the first time altered intra- and interregional synchronization of several cerebral networks related to tinnitus by combining resting-state ReHo, a measure of local synchrony, and seed-based functional connectivity analyses. We detected abnormal ReHo and functional connectivity within specific brain regions belonging to the attention network and the DMN. Furthermore, the abnormalities involved in the attention network showed significant correlations with the tinnitus distress.

### 4.1. Effects of Tinnitus on GM Volume

Subjective tinnitus is generally associated with hearing loss which decreases the neural output of the cochlea [33]. Peripheral damage, in turn, is believed to trigger aberrant neuroplastic changes in the CNS that result in tinnitus [10, 11]. However, some tinnitus patients have normal hearing out to the extended high frequencies and no obvious signs of hearing pathologies raising questions about the source of the aberrant neural activity involved in their phantom percepts [34]. To address this issue, we obtained structural and functional imaging data from normal controls and tinnitus patients, both with normal hearing thresholds out to the extended high frequencies and no evidence of hyperacusis or other medical problems. Thus, the tinnitus patients evaluated in this study may represent a somewhat unique tinnitus phenotype different from others with mild hearing loss or hyperacusis [35, 36].

Since tinnitus has been associated with structural changes, we compared GM and WM volumes but did not detect any differences between our normal hearing tinnitus patients and matched controls. Previous studies have reported decreases in GM volume in tinnitus patients in several brain regions including ACC, nucleus accumbens, ventromedial prefrontal cortex, inferior colliculus, hippocampus, superior frontal gyrus, occipital lobe, hypothalamus, and Heschl's gyrus [37–41]. Increases in GM volume have also been observed in tinnitus patients in the STG and MTG [41]. However, the changes in GM volume seen in these tinnitus patients were typically correlated with hearing loss particularly when testing was extended out beyond 8 kHz [37, 38, 41, 42]. Since we did not detect significant differences in GM volume between our tinnitus patients and controls, the most parsimonious explanation for this is the absence of any hearing loss out to 16 kHz and the absence of hyperacusis in our tinnitus patients. However, an alternative possibility is that our analytical techniques were not sensitive enough to detect regional differences in GM volume or intensity in our tinnitus patients [35, 41]. Regardless of which explanation is correct, our results suggest that abnormal functional neural networks can exist prior to major structural alterations in tinnitus patients with normal hearing.

### 4.2. Altered Intraregional Synchronized Activity (ReHo) in Chronic Tinnitus

In the present study, the ReHo is a data-driven method used to measure the extent to which brain activity is synchronized within a cluster of voxels, that is, neural synchronization within a specific brain region [22]. ReHo analyzes the regional connectivity of the neuronal tissue and may be seen as a measure of the smallest network integrity. Altered ReHo is possibly related to the changes of temporal aspects of the spontaneous neuronal activity in the regional brain. Therefore, increased or decreased ReHo reflects the local destruction of the synchronization of spontaneous neuronal activity in certain regions and implies functional deficits.

Increased local synchrony, as reflected in ReHo, could be a critical factor in initiating tinnitus since it might reflect the increased coherence of spontaneous neuronal activity [22, 43]. Enhanced local synchrony, or increased ReHo, could develop within a small cluster of neurons because of increased coupling of local factors such as loss of local intracortical inhibition, increased local intracortical excitation, aberrant receptors, and anatomical rewiring or plasticity-induced experience [44–51]. Local synchronization in one region of the brain could entrain other areas with preexisting long-range connections and reciprocal feedback circuits could further enhance interregional coupling [1, 52–54]. In some models, enhanced local synchrony results from inhomogeneities in cortical circuitry induced by cochlear hearing loss [53]. These models would be difficult to reconcile with the absence of hearing loss in our patients. However, certain cochlear pathologies involving damage to the inner hair cells or auditory nerve fibers can go undetected by PTA pointing out the need for more sophisticated testing to detect hidden hearing loss [55, 56].

In tinnitus patients, ReHo values were significantly enhanced in four regions, the left and right AI, left IFG, and right SMG. The AI and the IFG are key nodes in the attention networks, specifically the ECA network. On the basis of resting-state quantitative electroencephalography (qEEG), the AI and IFG have been implicated in tinnitus and specific tinnitus characteristics [57–59]. Moreover, tinnitus questionnaire scores were correlated to heart rate variability markers and related to bilateral neural activity in AI [57]. Greater synchrony of alpha activity was observed bilaterally in the AI of patients with more severe tinnitus-related distress [60]. AI, which serves in the cingulo-opercular network as well as in the attention network, exhibits altered intra- and interregional synchronization in a number of pathological situations [61–63]. Similarly, the vital role of the frontal cortex in subserving tinnitus mechanism has been postulated [1, 45, 52]. Previous research has already shown that the IFG is important for emotional processing of sounds [64, 65]. The IFG serves as the core region of response inhibition and IFG activity might mirror the attempt to control the bottom-up attention allocation to the tinnitus percept in a top-down manner [66]. Thus, the IFG and the AI play important roles in the top-down modulation of automatic or peripheral physiological responses to emotional experiences [67, 68]. Previous positron emission tomography (PET) imaging studies found significant correlation of brain glucose metabolism in the AI and IFG with the distress and/or duration of tinnitus or tinnitus-like sounds [69–71]. Taken together, these results suggest that tinnitus distress, salience, or attentional focus is associated with increased intraregional synchronization combined with enhanced functional interconnectivity in these cerebral networks.

We also found increased ReHo values in the SMG, a region also associated with the attention network [72, 73]. Prior EEG or PET studies indicate that the SMG may be involved in tinnitus [74–76]. Schmidt et al. demonstrated that the dorsal attention network, with seed regions in the bilateral intraparietal sulci, showed decreased correlations with the right SMG in tinnitus subjects [20]. Thus, the increased local synchronization in the SMG of tinnitus patients may alter the connectivity in the dorsal attention network. These features suggested that the alterations of the spontaneous fMRI BOLD signals in attention network might be due to the abnormal local synchronization in chronic tinnitus.

By contrast, the left cuneus showed reduced ReHo values in tinnitus patients, which may be due to compensatory mechanisms in visual regions associated with hearing a phantom sound. It has been suggested that sensory deprivation in the auditory modality could affect the function of the auditory modality [77, 78]. We speculated that the decreased ReHo may be due to increased attention devoted to processing a phantom sound which decreases neural synchronic with the visual system. Decreased spontaneous neural activity, as reflected in ALFF values, was also observed in visual areas [21], which again could be due to the increased salience of tinnitus. Anyhow, it is not appropriate to conclude from the relationships between decreased ReHo and dysfunction of the visual area in tinnitus. Whether reduced ReHo simply indicates visual impairment in tinnitus is unclear and needs further investigation.

### 4.3. Altered Interregional Synchronized Activity (Functional Connectivity) in Chronic Tinnitus

We further investigated the interregional synchronized neural activity in tinnitus patients using only seed regions which exhibited high ReHo values, namely, left and right AI, left IFG, and right SMG. The functional connectivity networks from four ROIs were shown clearly on the three-dimensional brain graphs (Figure 2(c)). Remarkably, our study showed significantly enhanced functional connectivity especially within the ECA network including the AI, the IFG, and the MFG. The increased functional connectivity between bilateral AI and bilateral MFG was positively correlated with tinnitus distress. Many neuroimaging studies have confirmed the involvement of frontal cortex for tinnitus [45, 71]. Rauschecker et al. demonstrated structural and functional differences of ventromedial prefrontal cortex in tinnitus patients that were linked to tinnitus loudness, indicating that frontal cortex may contribute to certain perceptual features of tinnitus [79]. The frontoinsular cortex potentially provides executive control overswitching attention between tinnitus and other conditions [80–82]. The differences in tinnitus connectivity might reflect an adaptation to decrease the salience of phantom noises and maintain attention on nonauditory events. Burton et al. also confirmed that functional connectivity in areas of ECA network in tinnitus group was greater than that in the control group. This connectivity was positively correlated with activity in the auditory cortex [12]. Similar to Burton, we also found increased functional connectivity between the AI and the STG, the center of primary auditory cortex. The ITG, another temporal cortex related to auditory perception, showed aberrant functional connectivity to the AI and IFG. Schecklmann et al. reported that tinnitus distress was correlated positively with brain metabolism in bilateral ITG using PET imaging [71]. In our study, there also existed enhanced interregional synchronization between right SMG and left IFG and right OFC, indicating the disrupted attention network associated with chronic tinnitus.

Besides the attention networks, significantly increased functional connectivity to several DMN regions, such as the precuneus, ACC, and PCC, was also observed in tinnitus patients. The DMN, encompassing precuneus and mesiofrontal and temporoparietal junction areas, appears to have strong negative correlations with a network of brain regions commonly activated during the performance of goal-directed cognitive tasks termed the “task-positive” network [83]. Using seed-based, independent component analysis (ICA) or graph theory analysis, previous functional connectivity studies have found altered interregional synchronization between the DMN and attention network in tinnitus subjects [12, 20, 84]. Our study was inconsistent with reduced functional connectivity within the DMN in Schmidt's study as well as the lack of significant differences between the DMN and attention networks in Wineland's study [20, 85]. These discrepancies might be due to the methodological features unique to the current study such as the use of seed regions with high ReHo values, the use of patient with normal hearing up to 16 kHz, and elimination of subjects with hyperacusis.

### 4.4. Limitations

This current work was an exploratory study using high ReHo values to select seed regions for subsequent connectivity analysis and had several inevitable limitations. First, our sample size was moderate, 29 tinnitus patients and 30 normal hearing subjects. Increasing the sample size with our approach would increase our ability to make causal relationships between the altered intra- or interregional synchronization and tinnitus characteristics. Furthermore, though this study has attempted to minimize the amount of scanner noise with earplugs, we cannot completely prevent subjects from hearing some sound. The existence of scanner noise may make the internal sound of tinnitus less salient thereby reducing the differences in resting-state networks between tinnitus and control groups. However, this limitation applies to virtually all resting-state studies in the literatures. Nevertheless, this limitation should be taken into consideration while attempting to draw conclusions on resting-state fMRI results in studies involving tinnitus patients as well as studies involving auditory stimulation in general. Finally, participants with hyperacusis were excluded from the current study because tinnitus is often accompanied by hyperacusis that could cause resting-state pathological brain activation in tinnitus patients according to previous neuroimaging studies [86, 87]. Since the tinnitus subjects without hyperacusis could not represent all the chronic tinnitus patients, the role of chronic tinnitus accompanying hyperacusis should also be taken into account in future explorations.

## 5. Conclusions 

Our combined ReHo and functional connectivity analyses demonstrated significantly altered infra- and interregional synchronization mainly within the attention network and the DMN prior to structural changes in tinnitus patients. The functional abnormalities within the ECA network showed associations with the tinnitus distress. Thus, tinnitus can be regarded as the consequence of multiple resting-state networks involved in different aspects of tinnitus, such as auditory perception, attention, and affect. The identification of both local regions of aberrant resting-state activity by ReHo as well as aberrant interregional disturbances in brain connectivity could help unravel the complex cerebral networks subserving tinnitus and enhance our understanding of the pathophysiological mechanisms of this disorder.

## Figures and Tables

**Table 1:** Characteristics of the participants.

	Tinnitus patients	Healthy controls	*P* value
	(*n* = 29)	(*n* = 30)	
Age (year)	40.9 ± 10.5	46.2 ± 11.9	0.074
Sex (male : female)	16 : 13	15 : 15	0.691
Education (years)	10.9 ± 2.2	11.1 ± 1.7	0.665
Tinnitus duration (months)	39.5 ± 33.7	—	—
THQ score	103.5 ± 74.4	—	—

Data are expressed as Mean ± SD. THQ: Tinnitus Handicap Questionnaires.

**Table 2:** Comparisons of the brain volumes between the tinnitus patients and healthy controls.

	Tinnitus patients	Healthy controls	*P* value
	(*n* = 29)	(*n* = 30)	
Gray matter	581.2 ± 26.4	576.1 ± 22.1	0.423
White matter	531.6 ± 25.6	528.8 ± 25.4	0.680
Brain parenchyma	1112.8 ± 33.5	1105.0 ± 37.7	0.401

Data are expressed as Mean ± SD.

**Table 3:** Differences in ReHo between tinnitus patients and healthy controls.

Brain region	BA	MNI coordinates	*T* score	Voxels
		*x*, *y*, *z* (mm)		
L anterior insular cortex	13	−36, 24, 9	3.1674	48
R anterior insular cortex	13	33, 21, 9	3.2775	32
L inferior frontal gyrus	47	−57, 39, 6	3.2861	39
R supramarginal gyrus	40	33, −39, 42	3.5553	27
L cuneus	18	−3, −96, 0	−3.3198	446

The threshold was set at *P* < 0.01 (AlphaSim correction). BA: Brodmann's area; MNI: Montreal Neurological Institute; L: left; R: right.

**Table 4:** Abnormal functional connectivity of four seed regions in tinnitus patients compared with healthy controls.

Seed region	Brain region	BA	MNI coordinates	*T* score	Voxels
			*x*, *y*, *z* (mm)		
L anterior insular cortex	L middle frontal gyrus	10	−39, 54, 0	3.4091	31
	R inferior temporal gyrus	20	60, −42, −18	3.4815	27
	R precuneus	7	21, −66, 39	4.1773	28

R anterior insular cortex	R middle frontal gyrus	11	12, 54, −6	4.6623	99
	R superior temporal gyrus	22	69, −21, 0	3.9777	26
	L precuneus	19	−30, −78, 33	2.9759	23
	L posterior cingulate cortex	29	−3, −51, 9	3.4933	40

L inferior frontal gyrus	R middle frontal gyrus	11	30, 42, −12	4.1975	100
	R inferior temporal gyrus	37	63, −54, −12	3.9566	131
	R anterior cingulated cortex	32	15, 30, 21	4.2129	27

R supramarginal gyrus	L inferior frontal gyrus	46	−45, 27, 21	3.6893	27
	R orbitofrontal cortex	47	48, 48, −15	4.0962	20

The threshold was set at *P* < 0.01 (AlphaSim correction). BA: Brodmann's area; MNI: Montreal Neurological Institute; L: left; R: right.
